# Relationship between anxiety, depressive symptoms and compulsive overeating disorder in patients with cardiovascular diseases

**DOI:** 10.1590/1518-8345.2567.3040

**Published:** 2018-09-03

**Authors:** Géssica Damares Garcia, Daniele Alcalá Pompeo, Letícia Palota Eid, Cláudia Bernardi Cesarino, Maria Helena Pinto, Laryssa Wilson Paiva Gonçalves

**Affiliations:** 1 Child and Adolescent Health Multidisciplinary Residency Student, Faculdade de Medicina de São José do Rio Preto, São José do Rio Preto, SP, Brazil.; 2 PhD, Adjunct Professor, Faculdade de Medicina de São José do Rio Preto, São José do Rio Preto, SP, Brazil.; 3 PhD, Adjunct Professor, Unidade Acadêmica Ciências da Saúde, Universidade Federal de Goiás, Jataí, GO, Brazil.; 4 MSc.

**Keywords:** Binge-Eating Disorder, Anxiety, Depression, Cardiovascular Diseases, Obesity, Health Evaluation

## Abstract

**Objectives:**

to identify the presence of compulsive overeating disorder in patients with cardiovascular diseases and to verify its relation with sociodemographic, clinical variables and the presence of anxiety and depressive symptoms.

**Method:**

cross-sectional, correlational study with a sample of 111 patients with cardiovascular diseases. The presence of anxiety and depressive symptoms was assessed by the *Hospital Anxiety and Depression Scale* instrument and compulsive overeating disorder was assessed through a likert instrument called the Periodic Eating Disorder Scale (Binge Eating Scale).

**Results:**

there was a predominance of patients without compulsive overeating disorder (n=91, 82%), followed by moderated compulsive overeating (n=15, 13.5%) and severe (n=5, 4.5%) associating to high levels of body mass index (p=0.010) and the presence of anxiety (p=0.017).

**Conclusion:**

Compulsive overeating disorder was present in 18% of the patients, being associated with body mass index and anxiety, suggesting that health professionals should pay attention to the comprehensive evaluation of patients with cardiovascular diseases. Important results emerged from this study, emphasizing the need to implement programs to improve the patients’ mental and physical health in both primary and specialized care services.

## Introduction

Recent statistics from the *American Heart Association* have shown that cardiovascular diseases (CVD) continue to lead the most common causes of mortality in the world. In 2013, they accounted for about 17.3 million of a total of 54 million deaths or 31.5% of all global deaths, with most, about 80%, occurring in low- and middle-income countries, including Brazil. Although mortality rates from these causes have declined, it is estimated that by 2030, 43.9% of the adult population in the United States should have some of its clinical forms^1^.

The cardiovascular diseases can, mostly, be prevented by combating traditional risk factors such as smoking, sedentarism, obesity, hypertension, inadequate diet and dyslipidemia^1^. In addition, recent studies have pointed out that psychological factors, such as emotional states of anxiety and depression, are also associated with the presence of these affections and, therefore, should be considered in their clinical management^1^ ^-^ ^3^.

A large cohort in the United Kingdom, recently, demonstrated that depression was prospectively associated with heart, cerebrovascular and peripheral diseases^2^. Similarly, the *American Heart Association* has assembled several evidences on the effect of anxiety, depressive symptoms, and other negative emotional states that act independently on the onset and worsening of cardiovascular diseases such as stroke and acute myocardial infarction^1^.

In addition to the symptoms of anxiety, depression, stress, sadness and anger, which are conditions frequently associated with these diseases, compulsive overeating can also be a coping strategy used to soften or solve everyday problems, providing a sense of pleasure^4^. 

This behavior characterized by the ingestion of high amounts of food in a delimited period, accompanied by sensation of loss of control is denominated compulsive overeating disorder^5^ ^-^ ^6^. To characterize the diagnosis, these events should occur at least two days per week in the last six months, associated with some loss of control characteristics and not accompanied by compensatory behaviors directed towards weight loss^5^ ^,^ ^7^.

Compulsive overeating comprises at least two elements: the subjective (feeling of loss of control) and the objective (amount of food consumption). An example of a situation that can trigger this disorder is the stress, with consequent cortisol release, which takes action stimulating food intake and weight gain^8^.

In the United States, a representative sample of adults from the *National Health and Wellness Survey* was recruited into an online panel to respond to an Internet survey regarding issues to assess compulsive eating behaviors. Out of the total of 22,397 interviewees, 344 reported criteria that met the diagnosis of compulsive overeating. The compulsive overeating frequency occurred, on average, in more than two or three days per week, with duration lasting 12 months and was characterized as severe symptoms^9^.

Clinically, compulsive overeating has often been associated with metabolic deficits and clinical conditions predisposing to the onset of cardiovascular diseases, such as obesity, dyslipidemia, and diabetes. The evidences suggest that this disorder, along with other eating disorders, may affect about 40% of people with type 2 diabetes *mellitus*, compromising the metabolic control and raising the risk of vascular complications^10^. However, individuals with compulsive overeating had a higher risk, expected in 10 years, of developing cardiovascular diseases, based on the Framingham risk score^11^.

Despite consistent evidence, this eating disorder is not investigated or evaluated effectively by professionals working in direct care. A study with patients diagnosed with heart failure revealed that problems of emotional origin are not adequately identified by health professionals, pointing to some probable reasons for this situation, such as difficulty for patients to reveal their emotional state, because of the fear of being labeled as mental illnesses carriers and the assistance given by professionals guided by the biomedical model, whose attention is mainly focused on the pathology and treatment^3^.

A recent study that aimed to investigate the knowledge and attitudes of Australian health professionals regarding compulsive overeating disorder, concluded that the professionals were reluctant to diagnose this disorder and the obesity as comorbid conditions. In addition, the knowledge of the physical complications associated with compulsive overeating came out as limited^12^.

The literature demonstrates the need for an approach focused on compulsive overeating symptoms as part of risk reduction programs and guidelines in clinical care for patients with cardiovascular diseases, as well as the identification and treatment of other stressful conditions of a psychological nature, such as anxiety and depression^4^. It is invaluable that the professionals are trained and sensitized to provide comprehensive care, considering the magnitude of psychological factors in several pathological processes. 

It is noted a scarce scientific production regarding the prevalence and factors associated with compulsive overeating in patients with cardiovascular diseases. Thus, the objective of this investigation was identifying the presence of compulsive overeating in patients with cardiovascular diseases and verifying its relation with sociodemographic, clinical variables and the presence of anxiety and depression symptoms.

## Method

This is a cross-sectional study, developed in a Clinical and Surgical Admission Unit designated for the cardiovascular specialty, of a Teaching Hospital in the State of São Paulo. The hospital is a large public institution, covenanted with 102 municipalities in the Northwest region of the State of São Paulo, with a total of 597 beds and 89,025 monthly visits.

The population consisted of patients with cardiovascular diseases admitted to the referred hospital, regardless of gender, aged 18 years or over and classified as overweight or obese. The exclusion criteria were: unable to communicate verbally and those who did not have cognitive conditions that would allow participation in the study, verified through the ability to inform their age or date of birth, residential address, day of the week and month. Participants who met the inclusion criteria were selected by means of consecutive and non-probabilistic sampling (n=111) and the data collection was performed in the period from 06/01/2015 to 02/28/2016.

Socio-demographic and clinical data were collected through a specific instrument, consisting of the following items: gender, age, marital status, education, current profession, income, body weight, height, body mass index, recent weight loss or gain, medical diagnosis and presence of comorbidities, such as hypertension, *diabetes mellitus*, dyslipidemias or other diseases.

The presence of anxiety and depressive symptoms was evaluated by the Hospital Anxiety and Depression Scale (HADS)^13^, in its Portuguese validated version^14^. The scale has 14 items (seven for the evaluation of anxious symptoms and seven for depressive symptoms), evaluated on a scale from zero to three points, with scores varying from zero to 21 points. Higher values indicate high symptomatology of anxiety and depression^14^.

Compulsive overeating was evaluated through a likert instrument named *Escala de Compulsão Alimentar Periódica* ECAP (Binge Eating Scale - BES).

The ECAP has 16 items and was developed specifically for the evaluation of obese individuals^15^, contemplating aspects related to the behavioral characteristics (e.g. amount of food that was consumed), emotional and cognitive. Each item has three to four possible answers, with values from zero (absence) to three (maximum severity), which must be selected according to the response that represents the individual. The score ranges from zero to 46 points. The individuals are classified according to the following scores: ≤17 = absence of compulsive overeating; 18 to 26 = moderate compulsive overeating; ≥27 = severe compulsive overeating.

As for its psychometric properties, ECAP presented internal consistency, measured by the Cronbach’s Alpha, of 0.85, considered moderately high^15^.

The ECAP was translated and adapted into the Portuguese language in 2001 and it was considered adequate for clinical use^16^.

The body mass index (BMI) was calculated by dividing weight by height squared. Overweight individuals were defined by the BMI of 25 kg/m^2^ to 29.9 kg/m^2^ and obesity by the BMI equal to or greater than 30 kg/m^2(^ ^17^.

During the hospitalization, the census of the hospitalized individuals in the Cardiovascular Unit was evaluated for the identification of the patients with cardiovascular diseases, by the researcher. Patients who met the inclusion criteria were invited to participate in the study and, after obtaining agreement, the researcher applied the sociodemographic characterization instruments, HADS and ECAP, through an interview, in a single moment.

The data were processed and analyzed through the *Statistica lPackage for Social Science* (SPSS®) version 19 for Windows IBM Company Copyright 2010. For the descriptive analysis of the data, measures of position (mean and median) and variability (standard deviation) were used. The internal consistency of the ECAP items was verified by Cronbach’s alpha. Qualitative data were associated with the use of the chi-square test. Quantitative data were compared using the student t test (for independent samples), ANOVA, Kruskall-Wallis and Mann-Whitney tests. The Pearson correlation test was used to analyze the normality of the continuous variables. The distribution of normality of the variables was investigated by the Kolmogorov-Smirnov test. The level of significance was set at 0.05.

This study was approved by the Research Ethics Committee of the School of Medicine of São José do Rio Preto, protocol number 1,059,880, on 12/05/2015.

## Results

Out of the 111 patients with cardiovascular diseases, 59 (53.2%) were males and 52 (46.8%) were females, ranging from the age of 34 to 85, mean age of 61.5±10.1 and median of 62.0. Most reported having a partner (n=76; 68,5%) having incomplete elementary school as maximum education (n=88, 79.3%), being retired (n=70, 63.1%) and having a monthly income of one to three minimum wages (n=97, 87.4%).

Coronary heart disease was the medical diagnosis of 93 patients (83.8%), followed by congestive heart failure (n=18, 16.2%). Most of the subjects reported having comorbidities associated with heart disease (27.0% had a comorbid condition, 34.2% had two comorbidities, and 33.3% had three comorbidities). The BMI ranged from 25.1 to 40.8 kg/m², with an average of 30.5 and a standard deviation of 3.7 kg/m². The majority of the patients reported not having gained or lost weight in the last six months (75.7% and 53.2%, respectively).

The anxiety symptom ranged from zero to 21 points, with an average of 8.8±4.6 points. The depressive symptomatology obtained a range from zero to 17 points, mean 5.8±4.2 points.

**Figure 1:**
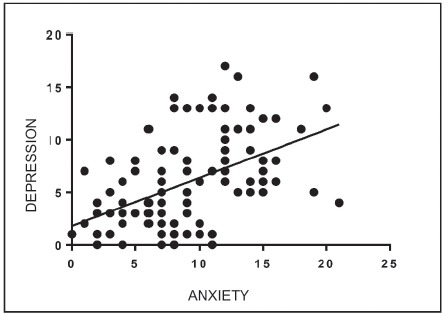
Pearson correlation analysis of the variables depression and anxiety (n=111). São José do Rio Preto, São Paulo, Brazil, 2015-2016

The results showed the presence of correlation of anxiety with age (r=-0.260, p=0.005) and depression (r=0.506; p=<0001), of weak and moderate magnitude, respectively. Figure 1 shows the correlation between anxiety and depression. There was no statistically significant connection between anxiety with gender (p=0.2119), medical diagnosis (p=0.5430) and BMI (p=0.2988).

Female participants presented higher depressive symptoms when compared to males (p=0.0182). Age (p=0.1549), medical diagnosis (p=0.1409) and BMI (p=0.707) did not influence the depressive symptoms.

In this study, patients without compulsive overeating predominated (n=91, 82%), followed by moderate (n=15, 13.5%) and severe (n= 5, 4.5%). The values ranged from zero to 29 points (range: zero to 46 points), with a mean of 8.34 ± 8.1 points. The internal consistency of ECAP, measured by Cronbach’s alpha coefficient, was 0.88.

The presence of compulsive overeating was associated with elevated BMI (p=0.010). The anxiety symptoms did not present significant correlation with compulsive overeating when classified as absent, moderate and severe (p=0.053); However, the results showed an association when the variable was dichotomized in the presence or absence of compulsive overeating (p=0.017), according to Table 1.

**Table 1:** Measurements of body mass index and anxiety symptoms variables according to participants’ compulsive overeating (n = 111). São José do Rio Preto, SP, Brazil, 2015-2016

Variables	Body Mass Index	Anxious Symptoms
	Mean [SD* (Median)]	Mean [SD* (Median)]
Compulsive overeating		
Absent (n=91)	30.00 [3.50 (29.390)]	8.34 [4.44 (8.00)]
Moderate (n=15)	33.28 [4.10 (33.590)]	11.33 [5.38 (12.00)]
Severe (n=5)	31.92 [4.49 (32.540)]	10.2 [3.76 (11.00)]
P-Value	0.010^†^	0.053^‡^
Compulsive overeating		
Absent (n=91)	--	8.34 [4.44 (8.00)]
Present (n=20)	--	11.05 [4.95 (11.5)]
P-Value	--	0.017^‡^

*standard deviation; †Analysis of Variance Test (ANOVA); ‡Kruskal-Wallis test

There were no associations between the presence of compulsive overeating and the variables: gender (p=0.286), education (p=0.970), presence of partner (p=0.899), medical diagnosis) and depression (p=0.497).

## Discussion

Compulsive overeating disorder endangers the physical and psychosocial health of the individuals. The results of this study showed that this behavior was present in 18% of the patients with cardiovascular diseases, being associated with elevation of BMI and anxiety. There was a prevalence of 8.4% in obese patients admitted to a cardiovascular rehabilitation unit in Croatia^4^, 16% in a sample of 5175 obese adults who sought for a weight reduction program^18^ and 12.2% in a sample of patients with type 2 diabetes *mellitus* ^19^.

A multi-centered study conducted by professionals from several countries with the objective to assess the epidemiological situation of compulsive overeating worldwide, through data from the World Health Organization, showed that its prevalence might range from 0.2% (Romania) to 4.7% (Brazil) in the population and that this percentage may increase in females, with a high body mass index, aged from 18 and 29 years old and that have musculoskeletal diseases, chronic pain, diabetes, hypertension, ulcers and headache . The prevalence of compulsive overeating in hypertensive patients was 1.8% and in patients with heart diseases was 0.9%^6^. 

In our sample, the prevalence of compulsive overeating was high (18%) when compared to the general population of Brazil (4.7%)^6^. Compulsive overeating may have been influenced by the increased anxiety and emotional distress of these people in dealing with the condition of having a chronic disease. We also emphasize that our sample was consisted of overweight and obese patients, whose rates tend to be higher.

It is noted a growing number of scientific investigations that demonstrated a strong association between this disorder and the presence of metabolic syndrome, obesity and diabetes^10^ ^,^ ^20^ ^-^ ^23^, which are conditions related to cardiovascular disease and that are constituted by predisposing factors for hypertension, endothelial dysfunction and dyslipidemia, that may trigger a major cardiovascular event, such as acute myocardial infarction.

In addition, this disorder was recently related to high concentrations of low-density lipoprotein cholesterol (LDL-cholesterol) in young women, in a large Japanese cohort^24^. These results reinforce the fact that individuals with this disorder have a high probability of having underlying metabolic and circulatory comorbidities.

A study based on data from the World Mental Health Surveys (comprising 19 countries and 52,095 adults) evaluated associations between the first onset of mental disorders and subsequent onset of hypertension. Depression, panic disorder, social phobia, specific phobia, compulsive overeating disorder, bulimia, alcohol and drug abuse were significantly associated with subsequent diagnosis of hypertension^20^.

Another cross-sectional study, which used data from the Brazilian Longitudinal Study of Adult Health (ELSA-Brazil) to investigate the relationship between recurrent episodes of compulsive overeating, nutritional profiles and lifestyle of its participants, showed that recurrent episodes of compulsive overeating were associated with obesity, overweight, female gender, age between 34 and 54, alcohol intake and sedentary behavior^25^, conditions characterized, classically, as risk factors for cardiovascular disease. It is believed that these results may explain, in part, the important prevalence of compulsive overeating in patients with cardiovascular diseases found in this study.

Although these evidences are consistent in the literature, a recent review emphasized the underdiagnosis of compulsive overeating disorder, either by not recognizing this condition as a distinct disorder or by lack of awareness among patients that the disorder is an abnormal behavior and amenable to treatment. The authors also showed that physicians only focus on the treatment of comorbidities such as diabetes, obesity, cardiovascular diseases, dyslipidemias, not considering the possible association of these conditions with compulsive overeating, therefore, reaching incomplete therapeutic results^26^.

Another relevant finding that deserves attention is the prevalence of increasing compulsive overeating at early ages. Results from a meta-analysis indicated that compulsive overeating was prevalent in more than a quarter of overweight and obese children and adolescents^27^, clearly demonstrating that preventive actions should be initiated as early as in childhood, so as to prevent future negative outcomes and to achieve positive impacts on morbimortality rates.

Similarly to our study, other studies have found an association between compulsive overeating and BMI elevation^28^ ^-^ ^29^. Obesity has multiple etiologies and the emotional factors seem to play a relevant role in its genesis. Conflicts and feelings of loneliness were significantly associated with eating disorders, mainly to compulsive overeating disorder, in severe obese patients^29^.

Although it is clear that there is a strongly positive association between binge eating and high BMI^18^ ^,^ ^22^, there are divergent findings in the literature regarding the mechanisms of action of compulsive overeating in the elevation of cardiovascular risk factors. There is no consensus as to whether this disorder acts independently or its effects are mediated by an increase in BMI. This brings to light the need for further investigation to elucidate possible hormonal pathways and other mechanisms at play by conferring this additional risk.

A recent Swedish study performed with 5850 individuals diagnosed with compulsive overeating, which aimed at identifying the main existing somatic comorbidities, it found a strong association of compulsive overeating with diabetes and diseases of the circulatory system, regardless of the presence of obesity. Within the sample of individuals with compulsive overeating, it was found that the presence of obesity was associated with increased risk of respiratory, gastrointestinal and cutaneous disorders, but not with other diseases classes^22^. This observation reinforces that the increased risk for some diseases in individuals with this disorder, including components of the metabolic syndrome, is not simply due to the effects of obesity.

In addition, obese individuals with compulsive overeating had an unfavorable metabolic and inflammatory profile when compared to obese individuals without the eating disorder. The presence of compulsive overeating showed a strong association with significant higher percentages of body mass index, waist circumference, insulin resistance, body fat mass and a lower lean body mass. Furthermore, the obese group with compulsive overeating had significant lower levels of high-density lipoprotein cholesterol and higher levels of glycated hemoglobin, uric acid, and C-reactive protein. All these differences remained significant after adjusting for body mass index^28^.

On the other hand, the prevalence of compulsive overeating was associated with higher risk of hypertension, hypertriglyceridemia, decreased HDL, insulin resistance and metabolic syndrome, apparently mediated by increased BMI, among participants in the Framingham Heart Study with compulsive overeating. The eating disorder was strongly associated, independently, with hyperglycemia alone. In addition, these individuals had more visceral, subcutaneous and hepatic fat^11^.

A recent study reinforced the idea that compulsive overeating does not seem to be independently related to cardiometabolic risk factors, and this connection is mediated by the elevation of BMI^18^. It is worth noting that although the mechanisms associated with elevated cardiovascular risk in patients with compulsive overeating remain unclear, the literature is consistent on the clinical relevance of screening and treating this disorder to reduce the risk of developing obesity and cardiovascular disease^11^ ^,^ ^18^.

Our results also evidenced the presence of anxiety as a factor that influences compulsive overeating, corroborating results of other studies^4^ ^,^ ^30^. In a review of the literature, it was found that anxiety is an important factor in the development and maintenance of compulsive overeating, supported by a set of previous researches that illustrate not only a high simultaneity between these disorders, but also the ways in which anxiety might play a unique role in the genesis of compulsive overeating^31^.

Several other evidences point to the importance of emotional deficits and stressors in the development of compulsive overeating, which may explain, in part, the association of this variable with anxiety. Recent models of rats submitted to various episodes of stress, developed compulsive overeating and hyperphagia, completely imitating the behavioral and metabolic characteristics of human compulsive overeating^8^.

In addition, subjects with binge eating reported greater psychological deficits when compared to obese and normal weight controls^32^, suggesting emotional difficulties. A study that sought to evaluate the impact of psychological factors on the incidence of compulsive overeating demonstrated a unique connection of this condition with anxious symptoms, regardless of the presence of depressive symptoms^30^, evidencing anxiety as a key factor in the onset of compulsive overeating and should be included in clinical investigations of this disorder.

Although our study found no association between depression and compulsive overeating, we emphasize the positive correlation between anxiety and depressive symptoms, corroborating the literature regarding the close relationship between these variables^33^ and, awakening, the attention for further investigations.

In this perspective, a recent research has shown that individuals with a diagnosis of compulsive overeating disorder have an increased chance of developing anxiety, depression and cardiovascular disorders, increased functional impairment and reduced quality of life, comparing to those without the diagnosis^33^, making it clear that comprehensive treatments must address the psychological antecedents, that are critical to the syndromic nature of this condition.

Considering that compulsive overeating can lead to unfavorable metabolic and circulatory profiles, nurses who work in direct care for patients with cardiovascular diseases should investigate the presence of the manifestations of this disorder, especially when associated with obesity and anxious symptomatology, in order to plan a wide and individualized care, obtaining positive responses regardless the established therapy.

In addition, it is believed that nurses may not obtain satisfactory results when teaching a patient about diet-related lifestyle changes if there is compulsive overeating. Therefore, we emphasize the need for future studies that investigate if there is a relation between these variables, as well as randomized controlled trials to clearly define the long-term damage associated with this eating disorder.

A limitation of this study is the non-verification of some variables that could be associated with compulsive overeating: specific clinical and laboratory data, personal and family history related to mental conditions, as well as the ways the person may act in face of adversities. In addition, our study was cross-section, not allowing us to evaluate the patient in other phases of the cardiovascular disease.

## Conclusion

The average levels of anxiety and depression in the studied sample were, respectively, 8.8 and 5.8 points. Compulsive overeating was present in 18% of the patients being associated with high BMI values and anxiety, suggesting that health professionals should pay attention to the physical and mental evaluation of patients with cardiovascular diseases. Important results emerged from this study, emphasizing the need to implement programs to improve patients’ mental and physical health in both primary and specialized care services.

## References

[1] Benjamin EJ, Blaha MJ, Chiuve SE, Cushman M, Das SR, Deo R (2017). Heart Disease and Stroke Statistics-2017 Update: A Report From
the American Heart Association. Circulation.

[2] Daskalopoulou M, George J, Walters K, Osborn DP, Batty GD, Stogiannis D (2016). Depression as a Risk Factor for the Initial Presentation of
Twelve Cardiac, Cerebrovascular, and Peripheral Arterial Diseases: Data
Linkage Study of 1.9 Million Women and Men. PLoS One.

[3] Polikandrioti M, Goudevenos J, Michalis LK, Koutelekos J, Kyristi H, Tzialas D (2015). Factors associated with depression and anxiety of hospitalized
patients with heart failure. Hellenic J Cardiol.

[4] Pokrajac-Bulian A, Tkalcic M, Ambrosi-Randic N (2013). Binge eating as a determinant of emotional state in overweight
and obese males with cardiovascular disease. Maturitas.

[5] Bąk-Sosnowska M (2017). Differential criteria for binge eating disorder and food
addiction in the context of causes and treatment of obesity. Psychiatr Pol.

[6] Kessler RC, Berglund PA, Chiu WT, Deitz AC, Hudson JI, Shahly V (2013). The Prevalence and Correlates of Binge Eating Disorder in the
World Health Organization World Mental Health Surveys. Biol Psychiatry.

[7] Montano CB, Rasgon NL, Herman BK (2016). Diagnosing binge eating disorder in a primary care
setting. Postgrad Med.

[8] Razzoli M, Pearson C, Crow S, Bartolomucci A (2017). Stress, overeating, and obesity: Insights from human studies and
preclinical models. Neurosci Biobehav.

[9] Pawaskar M, Solo K, Valant J, Schmitt E, Nwankwo M, Herman BK (2016). Characterization of Binge-Eating Behavior in Individuals With
Binge-Eating Disorder in an Adult Population in the United
States. Prim Care Companion CNS Disord.

[10] García-Mayor RV, García-Soidán FJ (2017). Eating disoders in type 2 diabetic people: Brief
review. Diabetes Metab Syndr.

[11] Abraham TM, Massaro JM, Hoffmann U, Yanovski JA, Fox CS (2014). Metabolic characterization of adults with binge eating in the
general population: the Framingham Heart Study. Obesity (Silver Spring).

[12] Cain B, Buck K, Fuller-Tyszkiewicz M, Krug I (2017). Australian Healthcare Professionals’ Knowledge of and Attitudes
toward Binge Eating Disorder. Front Psychol.

[13] Zigmond AS, Snaith RP (1983). The hospital anxiety and depression scale. Acta Psychiatr Scand.

[14] Botega NJ, Pereira WA, Bio MR, Garcia C, Zomignani MA (1995). Psychiatric morbidity among medical in-patients: a standardized
assessment (GHQ-12 and CIS-R) made by ‘lay’ interviewers in a Brazilian
hospital. Soc Psychiatry Psychiatr Epidemiol.

[15] Gormally J, Black S, Daston S, Rardin D (1982). The assessment of binge eating severity among obese
persons. Addict Behav.

[16] Freitas S, Lopes CS, Coutinho W, Appolinário JC (2001). Translation and adaptation into Portuguese of the Binge-Eating
Scale. Rev Bras Psiquiatr.

[17] World Health Organization (2018). Obesity and overweight.

[18] Leone A, Bedogni G, Ponissi V, Battezzati A, Beggio V, Magni P (2016). Contribution of binge eating behaviour to cardiometabolic risk
factors in subjects starting a weight loss or maintenance
programme. Br J Nutr.

[19] Nicolau J, Simó R, Sanchís P, Ayala L, Fortuny R, Zubillaga I (2015). Eating disorders are frequent among type 2 diabetic patients and
are associated with worse metabolic and psychological outcomes: results from
a cross-sectional study in primary and secondary care
settings. Acta Diabetol.

[20] Stein DJ, Aguilar-Gaxiola S, Alonso J, Bruffaerts R, de Jonge P, Liu Z (2014). Associations between mental disorders and subsequent onset of
hypertension. Gen Hosp Psychiatry.

[21] Mitchell JE (2016). Medical comorbidity and medical complications associated with
binge-eating disorder. Int J Eat Disord.

[22] Thornton LM, Watson HJ, Jangmo A, Welch E, Wiklund C, von Hausswolff-Juhlin Y (2017). Binge-eating disorder in the Swedish national registers: Somatic
comorbidity. Int J Eat Disord.

[23] Olguin P, Fuentes M, Gabler G, Guerdjikova AI, Keck PE, McElroy SL (2017). Medical comorbidity of binge eating disorder. Eat Weight Disord.

[24] Nakai Y, Noma S, Fukusima M, Taniguchi A, Teramukai S (2016). Serum Lipid Levels in Patients with Eating
Disorders. Intern Med.

[25] Souza da Silva T, Bisi Molina MD, Antunes Nunes MA, Perim de Faria C, Valadão Cade N (2016). Binge eating, sociodemographic and lifestyle factors in
participants of the ELSA-Brazil. J Eat Disord.

[26] Citrome L (2017). Binge-Eating Disorder and Comorbid Conditions: Differential
Diagnosis and Implications for Treatment. J Clin Psychiatry.

[27] He J, Cai Z, Fan X (2017). Prevalence of binge and loss of control eating among children and
adolescents with overweight and obesity: An exploratory
meta-analysis. Int J Eat Disord.

[28] Succurro E, Segura-Garcia C, Ruffo M, Caroleo M, Rania M, Aloi M (2015). Obese Patients With a Binge Eating Disorder Have an Unfavorable
Metabolic and Inflammatory Profile. Medicine (Baltimore).

[29] Koski M, Naukkarinen H (2017). Severe obesity, emotions and eating habits: a case-control
study. BMC Obes.

[30] Rosenbaum DL, White KS (2015). The relation of anxiety, depression, and stress to binge eating
behavior. J Health Psychol.

[31] Rosenbaum DL, White KS (2013). The role of anxiety in binge eating behavior: A critical
examination of theory and empirical literature. Health Psychol Res.

[32] Kittel R, Brauhardt A, Hilbert A (2015). Cognitive and emotional functioning in binge-eating disorder: A
systematic review. Int J Eat Disord.

[33] Sheehan DV, Herman BK (2015). The Psychological and Medical Factors Associated With Untreated
Binge Eating Disorder. Prim Care Companion CNS Disord.

